# Suicide Communication on Social Media and Its Psychological Mechanisms: An Examination of Chinese Microblog Users

**DOI:** 10.3390/ijerph120911506

**Published:** 2015-09-11

**Authors:** Qijin Cheng, Chi Leung Kwok, Tingshao Zhu, Li Guan, Paul S. F. Yip

**Affiliations:** 1 HKJC Center for Suicide Research and Prevention, The University of Hong Kong, Hong Kong, China; E-Mails: chengqj@connect.hku.hk (Q.C.); raykcl@hku.hk (C.L.K.); 2 Institute of Psychology, Chinese Academy of Sciences, Beijing 100101, China; E-Mails: tszhu@psych.ac.cn (T.Z.); guanl@psych.ac.cn (L.G.); 3 Insititute of Computing Technology, Chinese Academy of Sciences, Beijing 100101, China; 4 Department of Social Work and Social Administration, The University of Hong Kong, Hong Kong, China

**Keywords:** suicide, social media, microblog, negative affectivity, personality, Chinese

## Abstract

*Background*: This study aims to examine the characteristics of people who talk about suicide on Chinese microblogs (referred to as Weibo suicide communication (WSC)), and the psychological antecedents of such behaviors. *Methods*: An online survey was conducted on Weibo users. Differences in psychological and social demographic characteristics between those who exhibited WSC and those who did not were examined. Three theoretical models were proposed to explain the psychological mechanisms of WSC and their fitness was examined by Structural Equation Modeling (SEM). *Results*: 12.03% of our respondents exhibited WSC in the past 12 months. The WSC group was significantly younger and less educated, preferred using blogs and online forums for expressing themselves, and reported significantly greater suicide ideation, negative affectivity, and vulnerable personality compared to non-WSC users. SEM examinations found that Weibo users with higher negative affectivity or/and suicidal ideation, who were also using blogs and forums more, exhibited a significantly higher possibility of WSC. *Conclusion*: Weibo users who are at greater suicide risk are more likely to talk about suicide on Weibo. WSC is a sign of negative affectivity or suicide ideation, and should be responded to with emotional support and suicide prevention services.

## 1. Introduction

Are people who talk about suicide really at risk of committing suicide? Would people with a suicide risk talk about suicide? (The behavior of verbally talking about suicide is hereafter referred to as *suicide communication*). These questions do not only concern suicide researchers but also healthcare practitioners and the general public. With the broad penetration of the Internet in daily life, such concerns also relate to the online environment. For researchers and professionals, online communication such as forums and microblogs (Weibo means “microblog” in Chinese) present a new arena to detect and reach out to suicidal individuals [1]. Some researchers have used suicide-related keywords to extract data from the Internet as a proxy of suicidal thoughts and behaviors [2,3]. Researchers are now working with data scientists to develop computer algorithms to estimate an online user’s risks of suicide or depression by examining the linguistic features of their social media posts [4,5]. However, a challenge to the usefulness of those studies is that it is not clear whether those at-risk Internet users are likely to express their true thoughts and feelings on social media or to conceal them. To answer this question it is important to estimate the extent to which online detection by linguistic clues may miss some at-risk users.

For lay people and Internet industry members, there is also increasing concern with what a proper response ought to be when someone talks about suicide on social media. A recent example is that of an American man who posted a hoax suicide threat on Facebook to test the platform’s suicide prevention program [6]. Similar cases have also happened in China. According to the latest survey in China, over half of the Chinese population uses the Internet and about 90% of all the Internet users are using at least one kind of online social media [7]. Weibo is the most popular social media site in China with more than 500 million registered users, and it is often reported by the mass media as a popular platform for individuals to “live broadcast” their suicidal behaviors [8]. There were unfortunate cases where individuals expressed suicidal thoughts on social media but received feedback that mocked them or showed indifferent attitudes toward them [8,9]. On the other hand, some suicide posts were reported by the media as a “hoax” because the police, after many hours of searching, found those who posted suicide content online were still alive and not self-harming [10,11,12]. Commentary articles, published in national media such as *People’s Daily* and *Xinhua News Agency*, criticized those hoax cases for making fun of other online users and abusing public resources and suggested punishing such behavior [10,13,14]. However, as far as we know, there is no empirical evidence on how much of the online sharing of suicide messages is actually a “hoax” or “abuse”. Filling this gap of knowledge would help address public concerns and inform lay users and social media administrators on decisions for proper responses.

Literature and textbooks of suicide research and prevention commonly emphasize that it is a myth to think people who talk about suicide are not really suicidal [15]. Western studies suggested that verbal expression of suicide should be interpreted as a sign of suicidal intention (hereafter referred to as “warning signs theory”) [16]. In the warning signs of suicide generated by the American Association of Suicidology, “talking about suicide” along with “threatening suicide” and “looking for ways to die by suicide” are listed as signs of acute suicide risk [16,17]. Raising the public awareness of suicide warning signs and gatekeeper training are therefore proposed as important strategies for crisis intervention and suicide prevention [16,18]. On the other hand, other studies argued that, under certain conditions, talking about suicide or even trying to harm oneself might be interpreted as a cry for help, which may not necessarily be associated with acute suicide risk but may reflect ambivalent struggles over negative affectivity and emotional instability (hereafter referred to as the “cry for help theory”) [19,20]. In psychological research, negative affectivity and emotional instability are found to be risk factors for suicide [21]. Therefore, we can combine the warning signs theory and the cry for help theory to determine that people who talk about suicide are at a certain level of risk. The inverse proposition is that people who are not at risk of suicide would not talk about suicide.

There are significant gaps to directly applying the warning signs theory or the cry for help theory to the online situation. Prior studies on suicide communication primarily collected their data by analyzing suicide notes or interviewing suicide attempters or family members of those who committed suicide [19,22]. However, there might be fundamental differences between people who committed suicide, attempted suicide, and those who had suicidal ideation but never took action in terms of how they talk about suicide and the underlying psychological mechanisms [20,23]. From the perspective of public health, early detection of people who exhibit risk factors for suicide, such as suicidal ideation and depression, would be more effective for providing intervention and reducing suicide rates than merely treating people who already attempted suicide and are at high suicide risk [24]. However, there is very limited study to examine among the broader population, not just among those who have committed suicide or attempted suicide and were hospitalized, whether individuals who talked about suicide are certainly at greater suicide risk than those who did not talk about it, and whether individuals at greater risk would be more likely to talk about suicide.

In view of the research gaps and the social significance of proper reactions to the online expression of suicide, the present study aims to provide empirical answers to the old questions in the context of Chinese social media: are those who are talking about suicide on Weibo (we refer to this kind of action as *Weibo suicide communication* hereafter) really at greater risk of suicide? We understand that some literature refers to *suicide communication* as particular communication associated with sequential suicide death, of which suicide notes are often studied [22,23]. However, we would like to clarify that the present paper refers to an act as *suicide communication* as long as the communication is about suicide, which may or may not be followed by suicidal behaviors. It is the uncertain prospect that allows possibilities for intervention.

The findings would be particularly meaningful if they help us understand suicide among youth, which is a significant public health problem in China and globally. Despite the overall suicide rates in China decreasing in the past decade, this trend was not observed in young males [25]. Suicide remains the leading cause of death for people aged 15–34 in China [26] and the second leading cause of death in 15–29-year-olds globally [27]. Meanwhile, youth also constitute the major body of social media users. In China, 68.2% of Weibo users are aged 30 or younger [7].

The findings would be particularly meaningful if they help us understand suicide among youth, which is a significant public health problem in China and globally. Despite the overall suicide rates in China decreasing in the past decade, this trend was not observed in young males [25]. Suicide remains the leading cause of death for people aged 15–34 in China [26] and the second leading cause of death in 15–29-year-olds globally [27]. Meanwhile, youth also constitute the major body of social media users. In China, 68.2% of Weibo users are aged 30 or younger [7].

### Theoretical Models

To find psychological antecedents of Weibo suicide communication (WSC), we proposed to test three theoretical models and compare their fitness with each other.

**Figure 1:**
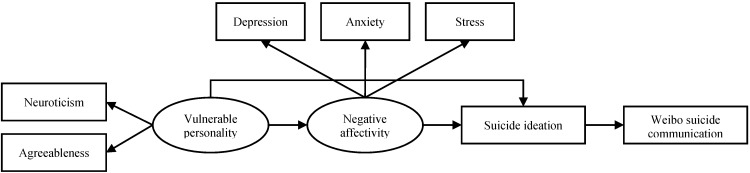
Model 1 based on the warning signs theory.

If the warning signs theory is applied, our first theoretical model assumes that WSC can be fully explained by suicidal ideation: people with suicidal ideation would exhibit WSC and WSC is a significant sign of suicidal ideation. There might be other antecedents of suicide risk that are also potentially related to WSC, but the relationship must be mediated by suicidal ideation (Figure 1). Here we propose that the potential antecedents of WSC include negative affectivity and vulnerable personality alongside suicidal ideation. States of negative affectivity, such as depression, anxiety, and stress, have been widely found to be associated with suicidal ideation [28,29,30,31,32,33]. As for personality traits, according to recent neuropsychiatric research, high neuroticism and low agreeableness have strong predictive values for a genetic vulnerability to depression [34]. Neuroticism is defined as a lack of affect and emotional control and is commonly found to be a predictor for depression, anxiety, and poor coping with stressors [35,36]. Neuroticism has also been consistently found to be associated with higher suicide risk in the general population [37,38]. On the other hand, findings on the relationship between agreeableness (which indicates a tendency to be trusting and cooperative) and depression or anxiety are not consistent [39]. Nonetheless, some studies found that people with low agreeableness were less social, less likely to cope by seeking support, and reported higher degrees of depression [40,41]. Therefore, we proposed a personality consisting of high neuroticism and low agreeableness to be vulnerable to depression and suicidal ideation. Furthermore, such a personality might be related to WSC via negative affectivity and suicidal ideation (Figure 1).

**Figure 2:**
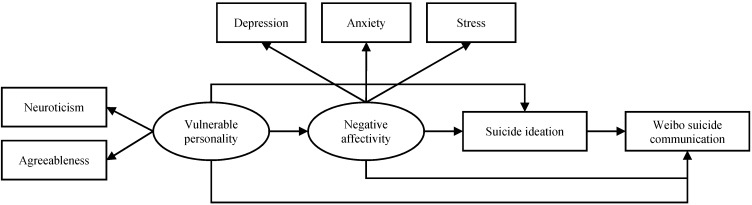
Model 2 based on the warning signs theory and the cry for help theory.

If the cry for help theory is also applied, the potential antecedents of suicidal ideation such as vulnerable personality and negative affectivity can also be connected to WSC directly, without the mediation of suicidal ideation. This second model is illustrated in Figure 2.

**Figure 3:**
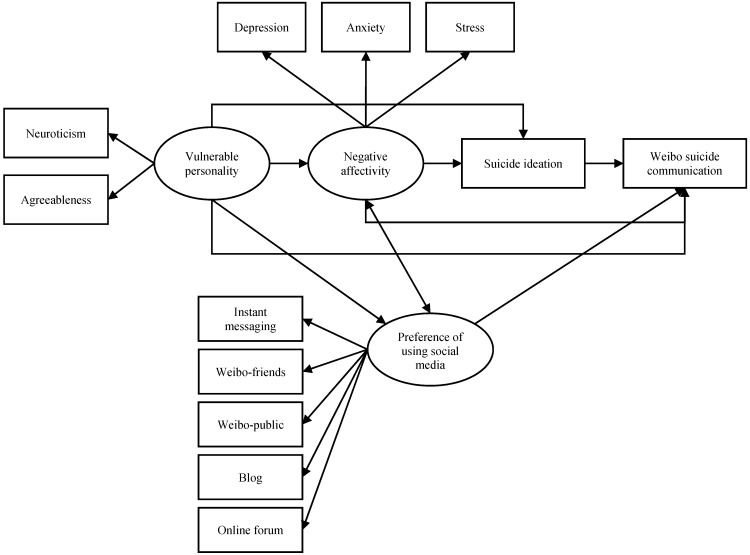
Model 3 based on the warning signs theory, the cry for help theory, and a preference for using social media.

In addition, since our focus is on investigating suicidal communication in an online environment, we would like to explore whether the preference for using social media to communicate also plays a role in the psychological mechanism of WSC. As far as we know, there is no publicly available research report suggesting a direct relationship between suicidal ideation and a preference for using social media. We might reasonably expect that people who prefer using social media in general would also have a higher chance of expressing suicidal communication on social media than those who do not. In addition, social media usage was suggested or found in some studies to be related with negative affectivity and vulnerable personality. A recent study on a large-scale representative sample of American youth found that neuroticism is positively associated with social media usage [42], which is consistent with other studies using smaller sample sizes [43,44,45,46,47]. Although few studies could identify a relationship between agreeableness and social media usage [43,48], one study on university students found a negative association between agreeableness and general usage of the Internet [48]. Therefore, we hypothesize that a vulnerable personality may lead to a greater preference for using social media and a higher chance of WSC. The relationship between negative affectivity, especially depression, and a preference for using social media has also been studied broadly but with inconsistent findings [41,49,50]. Nonetheless, a national longitudinal panel study on American youth found that heavy Internet use, including using social networking sites, was associated with an increase in depression, whereas increased depression also predicted greater use of the Internet [51]. Therefore, we hypothesize that greater negative affectivity is correlated with a greater preference for using social media and a higher chance of WSC.

Following a national survey on Chinese Internet development, we defined social media in China as online platforms that primarily serve social purposes, including instant messaging (e.g. WeChat, an application for chatting and social purposes, which combines some functions of WhatsApp and Twitter), microblogs (*i.e.*, Weibo), blogs, and online forums [7]. The framework is illustrated in Figure 3.

## 2. Methods

An online survey was conducted to examine online social media users’ profiles, mental health characteristics, and communication patterns in Mainland China.

### 2.1. Survey Procedure

The survey was conducted from May to July 2014. Participants were recruited through a widely distributed survey invitation on Weibo. The survey invitations were sent out through three approaches: (1) posted on the research team’s official Weibo account; (2) randomly sent to Weibo users through another registered non-official Weibo account; and (3) posted to the Weibo page of a psychology professor who has more than 970,000 followers and was invited to post the recruiting information. A more detailed report of the survey procedure can be found in one recent publication [4].

From the respondents, 1196 Weibo users started the survey and 1040 completed it. Because the online survey system did not allow a respondent to skip questions before submitting the questionnaire, there is no missing value in the 1040 completed questionnaires. After removing respondents from duplicate IP addresses, 989 completed cases remained for data analysis. Following the Checklist for Reporting Results of Internet E-Surveys (CHERRIES) [52], the response rate can be estimated as 82.7%.

### 2.2. Ethical Consideration

The study obtained ethical approval from the Institute Review Board of the Institute of Psychology at the Chinese Academy of Sciences (Ref #: H09036). Survey participants were restricted to those aged 18 and above (by self-report). An informed consent form had to be obtained from the participant online before filling in the questionnaire. The survey website was located on a server belonging to the Chinese Academy of Sciences. All the data was stored in a secured environment. The survey webpage contained a message that encouraged respondents to seek help if they felt distressed, and provided contact information for a crisis intervention hotline (800-810-1117) which is toll-free in China. An incentive was provided: upon completing the survey, 30 RMB credits would be added to his/her mobile phone.

### 2.3. Measurements

*Weibo Suicide Communication (WSC)*. The respondent was asked whether or not he/she had told others via Weibo in the past 12 months that he/she wanted to kill himself/herself. Respondents could choose among five options: *never*, *sometimes*, *about half the time*, *usually*, and *always*. When examining the characteristics of people who engaged in WSC, we dichotomized the five options as follows: WSC group (*sometimes to always*) and Non-WSC group (*never*). However, when examining the three candidate models, we retained the five categories so that the original data structure could be retained.

*Suicide ideation.* The suicide ideation sub-scale of the Chinese version of the Suicide Probability Scale (SPS) [53,54,55] was used to assess the respondents’ suicide ideation. The SPS was originally designed in English and then translated into Chinese. The Chinese version has shown good validity and reliability [56]. In particular, the suicide ideation sub-scale showed a significant correlation with Beck’s Scale of Suicide Ideation when testing among university students in China [56]. In our study, the Cronbach’s alpha coefficient was 0.834, showing a good level of internal consistency.

*Negative affectivity.* The Chinese version of the Depression-Anxiety-Stress Scale (DASS-21) [57] was used to measure the respondents’ negative affectivity. DASS-21 was designed to measure their negative affectivity as a whole, as well as in the three dimensions of depression, anxiety, and stress, and has been widely used in community surveys on the population’s mental status [58,59]. The Chinese version has been validated in China and has shown good construct validity and criterion-related validity [57]. In our study, the Cronbach’s alpha coefficient was 0.859 for the depression subscale, 0.767 for the anxiety subscale, and 0.821 for the stress subscale.

*Vulnerable personality*. The subscales of neuroticism and agreeableness in the Chinese version of the Big Five Personality Inventory [60,61] were used to assess respondents’ vulnerable personality (high neuroticism and low agreeableness). The Cronbach’s alpha coefficients were 0.739 and 0.680 for neuroticism and agreeableness, respectively. The internal consistency of the agreeableness subscale was marginally acceptable.

*Preference for using social media.* Categories of reporting the frequency of use included instant messaging, posting to Weibo within a circle of friends (a similar function as the Facebook timeline), posting to Weibo to the public (a similar function as Twitter), blogging, and the use of online forums. Respondents were asked to report how frequently they use the following five types of social media to express themselves in regular situations: *never use*, *occasionally use*, *use every week*, *use almost every day*, and *use frequently every day*.

*Demographics*. Respondents were invited to provide their profile information, including age, gender, education level, marital status, and living status (living alone, with family/partner, with friends or with others).

### 2.4. Data Analyses

SAS version 9.3 for Windows [62] and Mplus version 5 [63] were used for computing statistical analyses. The internal consistency of measures was assessed using Cronbach’s alpha coefficient [64]. We first examined the characteristics of respondents who had previously expressed suicidal thoughts on Weibo. Demographic characteristics and model variables were summarized using descriptive statistics. The Mann-Whitney-Wilcoxon test for continuous variables and the chi-square test for categorical variables were then applied to examine the difference between the WSC and Non-WSC groups.

Unlike other variables measured by well-established scales, the measurement of the preference for using social media was newly constructed by us. Exploratory factor analysis (EFA) was, therefore, used to determine the optimal number of common factors. As the multinormality of data was obviously not valid, the principal-axis factoring method was specified for factor extraction. Factors were rotated using orthogonal varimax rotation. Based on the rotated factor loadings, the association of the latent construct(s) with WSC was then examined. Composite reliability (CR) and average variance extracted (AVE) were also employed to evaluate the convergent validity (CR > 0.7; AVE > 0.5) and discriminant validity (square root of AVE > correlations between latent variables) [65,66]. This step resulted in a revised measure of preference for using social media.

We then moved to examine the fitness of the three proposed models. Spearman’s rank correlation was computed to show the association among model variables. After that, structural equation modeling (SEM) was conducted to evaluate the proposed models. A Multiple Indicators and Multiple Causes (MIMIC) model was used to adjust for demographic covariates, which were found to be significantly associated with WSC in the previous analysis. As all the proposed models involved an ordinal outcome variable (had *never* to *always* performed WSC), the robust weighted least squares estimator using a diagonal weight matrix was considered for parameter estimates with standardization using the variances of continuous latent variables and the variances of independent and outcome variables [63]. Direct and specific indirect effects would be reported and their statistical significances were evaluated by bootstrapping with 500 bootstrap samples [67].

The chi-square statistic of each proposed model was reported, but due to its high sensitivity to the sample size, it was not considered a reasonable assessment of model fit [68]. Two absolute fit indices, namely the root mean square error of approximation (RMSEA) and the weighted root mean square residual (WRMR), and two incremental fit indices, namely the comparative fit index (CFI) and the non-normed fit index (NNFI), were used to assess the goodness of fit of the proposed models. According to the suggestion by Hu and Bentler [69], a model having CFI > 0.95, NNFI > 0.95, and RMSEA < 0.06 indicates a good fit for the continuous outcome variables. Yu and Muthén [70] further showed that these cutoffs work reasonably well with categorical outcomes. In addition, a WRMR less than a cutoff value of 1.0 indicates a good fit of the model [71].

## 3. Results

### 3.1. Characteristics of the WSC Group

Among the 989 respondents, 119 (12.0%) reported WSC in the past 12 months, including 82 who reported *sometimes*, 25 *about half the time*, eight *usually*, and four *always*. We grouped the respondents into WSC and Non-WSC groups. Table 1 summarizes the demographic characteristics of the two groups. The two groups had no statistically significant difference in gender (${\text{χ}}^{2}$ = 0.232, d.f. = 1, *p* = 0.630) but did in education level (${\text{χ}}^{2}$ = 9.452, d.f. = 2, *p* = 0.009) and age (Mann-Whitney-Wilcoxon test: *p* = 0.010). Lower education attainment and younger age were associated with a higher chance of WSC. The results warranted us to adjust the two demographic characteristics in the model testing.

**Table 1:** Summary of demographic characteristics (% of column total) and statistical tests between groups.

Demographics	Weibo Suicide Communication			
	Total	Yes (*n* = 119)	No (*n* = 870)	*p* Value
**Gender**				0.630
Male	610	71 (59.7)	539 (62.0)	
Female	379	48 (40.3)	331 (38.0)	
**Education Level**				0.009
Vocation or below	98	18 (15.1)	80 (9.2)	
College/Undergraduate	759	94 (79.0)	665 (76.4)	
Master or above	132	7 (5.9)	125 (14.4)	
**Student**				0.971
Yes	575	69 (58.0)	506 (58.2)	
No	414	50 (42.0)	364 (41.8)	
**Marital Status ^2^**				0.636
In relationship/Married	360	48 (40.3)	312 (35.9)	
Separated/Divorced	9	1 (0.8)	8 (0.9)	
Single	620	70 (58.8)	550 (63.2)	
**Living Status**				0.095
Alone	120	18 (15.1)	102 (11.7)	
With family/partner	545	71 (59.7)	474 (54.5)	
With friend	158	10 (8.4)	148 (17.0)	
With others	166	20 (16.8)	146 (16.8)	
**Age ^1^**				0.010
Mean	24.2	23.1	24.4	
Standard deviation	4.9	3.4	5.1	

**^1^** No case reported widowed; **^2^** Excluded a respondent reporting age 100 (having communication of suicide).

Table 2 summarizes the statistics of the variables and items in the three candidate models and the associated Mann-Whitney-Wilcoxon tests between the two groups. The two groups did not show significant differences in preference for using instant messaging (*p* = 0.497), Weibo messaging with friends (*p* = 0.50), and Weibo posts broadcasting to the public (*p* = 0.076) for regular communication. However, the two groups showed significant differences in all other variables (all *p*-values < 0.05). Having higher degrees of suicide ideation, depression, anxiety, stress, and higher neuroticism but lower agreeableness are found to be significantly correlated with WSC. In addition, a higher preference for writing a blog and posting in an online forum is also correlated with WSC. The results supported further examination of the three candidate models.

**Table 2:** Means (standard deviation) of observed variables and items and Mann-Whitney-Wilcoxon tests between groups.

Measures	Weibo Suicide Communication				
	Range	Total	Yes (*n* = 119)	No (*n* = 870)	*p* Value
**Suicide Ideation**	8–32	11.5 (3.2)	14.5 (4.3)	11.1 (2.8)	<0.001
**DASS21**					
Depression	0–21	2.9 (3.5)	6.6 (5.0)	2.4 (3.0)	<0.001
Anxiety	0–21	4.1 (3.3)	6.9 (4.0)	3.7 (3.0)	<0.001
Stress	0–21	4.8 (3.8)	7.8 (4.6)	4.4 (3.5)	<0.001
**Personality**					
Neuroticism	8–40	25.1 (5.4)	27.3 (5.5)	24.9 (5.4)	<0.001
Agreeableness	9–45	32.9 (4.9)	30.1 (4.6)	33.3 (4.9)	<0.001
**Social Media Preference**					
Instant messaging	0–4	2.2 (1.1)	2.1 (1.0)	2.2 (1.1)	0.497
Weibo-friends	0–4	2.2 (1.1)	2.2 (1.0)	2.2 (1.2)	0.854
Weibo-public	0–4	2.0 (1.1)	2.2 (1.1)	2.0 (1.1)	0.076
Blogs	0–4	0.6 (0.9)	1.1 (1.1)	0.6 (0.9)	<0.001
Online forums	0–4	0.5 (0.8)	1.0 (0.9)	0.4 (0.7)	<0.001

### 3.2. Optimized Measure of Preference forUsing Social Media

Both the scree-plot and cumulative proportion of variance suggested that two common factors should be retained. Table 3 shows the factor loadings after orthogonal varimax rotation. The two factors, in total, could explain 45.9% of total variance. The first factor was composed of high factor loadings for the first three items (instant messaging: 0.496; Weibo-friends: 0.780; and Weibo-public: 0.760) but very low factor loadings for the other two (blogs: 0.190; and online forums: 0.095). The second factor, on the contrary, had high factor loadings for blogs and forums (0.636 and 0.624).

**Table 3:** Exploratory factor analysis of five-item preference for using social media.

Items	Factor 1	Factor 2	Communalities
Instant messaging	0.496	0.101	0.256
Weibo-friends	0.780	0.134	0.626
Weibo-public	0.760	0.221	0.627
Blogs	0.190	0.636	0.441
Online forums	0.095	0.624	0.398
Proportion of variance explained	28.9%	17.0%	-

Based on these rotated factor loadings, two latent constructs were developed. The first factor is composed of preference for instant messaging and Weibo, which is largely reflecting our respondents’ general profile as Weibo users. The second factor is composed of preference for blogs and online forums, which have a longer history than Weibo and different qualities from Weibo in terms of interaction dynamics. In fact, among the different types of social media, online forums have the longest history in China (e.g., Tianya Forum, one of the most popular online forums in China, was launched in 1999), followed by blogs (the first blog service provider in China was launched in 2003), whereas the first Weibo site in China was launched in 2005, and WeChat, a popular instant messaging application, was launched in 2010. Therefore, we labeled Factor 1 as “preference for using newer social media”, and Factor 2 as “preference for using more traditional social media”.

**Figure 4:**
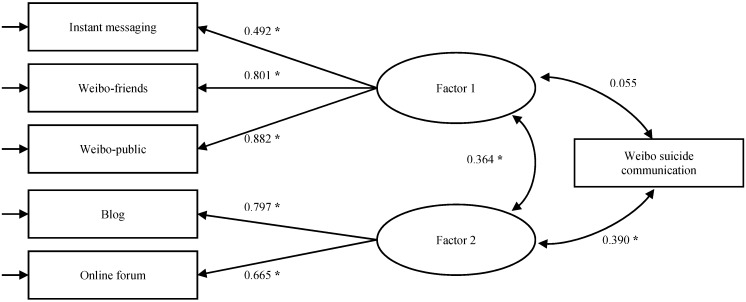
Association between latent constructs of five-item preference for using social media and Weibo suicide communication (* *p* < 0.01).

**Figure 5:**
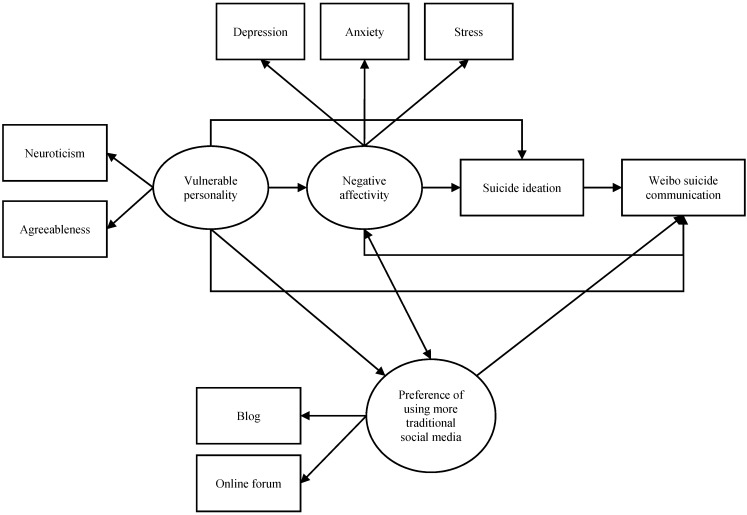
Model 3b, the revision of Model 3.

Figure 4 depicts a model testing the association of two latent constructs with WSC using Mplus version 5. The model showed a good fit, with ${\text{χ}}^{2}$ (6) = 20.6, *p* = 0.002, CFI = 0.966, NNFI = 0.961, RMSEA = 0.050, and WRMR = 0.612. The correlation between the two latent constructs was moderate (0.364, 95% C.I. = 0.288 to 0.439, *p* < 0.001). The composite reliabilities for Factor 1 and Factor 2 were 0.779 and 0.698, respectively. The average variance extracted was 0.554 for Factor 1 (square root = 0.744) and 0.539 for Factor 2 (square root = 0.734). These values demonstrate that both factors had adequate levels of reliability, convergent validity, and discriminant validity. However, only the second factor showed a significantly positive correlation with WSC (0.390, 95% C.I. = 0.299 to 0.481, *p* < 0.001), whereas Factor 1 reported a very weak association (0.055, 95% C.I. = −0.063 to 0.173, *p* = 0.365). Therefore, we revised Model 3 to Model 3b (Figure 5), only including Factor 2 as a latent variable of “preference for using more traditional social media.”

### 3.3. Theoretical Models

Table 4 shows the Spearman’s rank correlations (r) which firstly explore the direct relationship between each pair of model variables. Moderate to strong associations could be detected among WSC, suicide ideation, three subscales of DASS21, and two personality subscales ( |r| = 0.129–0.751, all *p*-values < 0.01). For the two items measuring preference for social media, their internal correlations were statistically significant (r = 0.456, *p* < 0.001), but their associations with other variables were relatively weak (|r| = 0.012–0.225). A moderate and significant positive correlation was found between using online forums and WSC (r = 0.225, *p* < 0.001), followed by using blogs and WSC (r = 0.182, *p* < 0.001), and using online forums and depression (r = 0.099, *p* = 0.002). The latter justifies the inclusion of a non-causal relationship between negative affectivity and preference for using social media in Model 3b. Since our previous analysis found that WSC is significantly correlated with education level and age, we controlled these two variables as covariates in model fitting.

**Table 4:** Spearman’s rank correlation.

No.	Model Variables	1	2	3	4	5	6	7	8	9
1	Weibo suicide communication	1								
2	Suicide ideation	0.276	1							
3	Depression	0.307	0.551	1						
4	Anxiety	0.284	0.492	0.661	1					
5	Stress	0.254	0.472	0.671	0.751	1				
6	Neuroticism	0.129	0.494	0.544	0.549	0.601	1			
7	Agreeableness	−0.225	−0.315	−0.384	−0.365	−0.431	−0.449	1		
8	Blogs	0.182	0.027	0.012	0.020	0.023	−0.069	0.018	1	
9	Online forums	0.225	0.078	0.099	0.063	0.045	−0.036	−0.093	0.456	1

SEM results showed that Model 1 had a poor fit as indicated by three out of four fit indices (CFI = 0.923, NNFI = 0.923, RMSEA = 0.088, and WRMR = 0.859; ${\text{χ}}^{2}$ (12) = 104.5, *p* < 0.001), although all parameter estimates from the structural part were statistically significant. The first model was not supported. Model 2 had a moderate but still not good fit (CFI = 0.953, NNFI = 0.949, RMSEA = 0.072, and WRMR = 0.695; ${\text{χ}}^{2}$ (11) = 67.6, *p* < 0.001).

**Figure 6:**
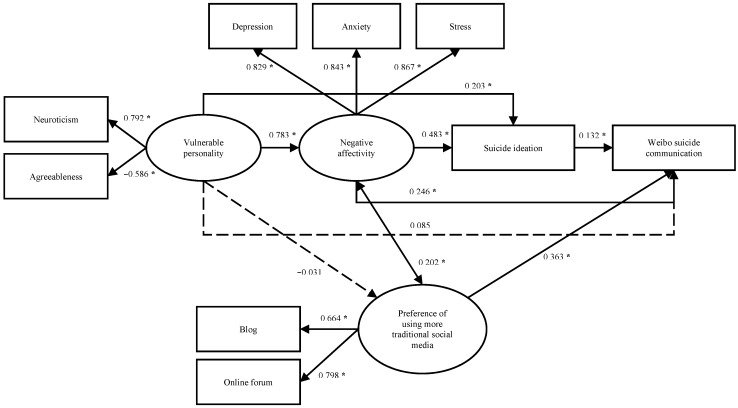
Results of SEM on Model 3b (age and education level were included in the model as covariates, * *p* < 0.05).

Model 3b showed a good fit to the data, with ${\text{χ}}^{2}$ (17) = 67.6, *p* < 0.001, CFI = 0.964, NNFI = 0.960, RMSEA = 0.055, and WRMR = 0.709. Results showed that all estimated path parameters were statistically significant (Figure 6), except for the paths from vulnerable personality to WSC (0.085, 95% C.I. = −0.144 to 0.313, *p* = 0.468) and from vulnerable personality to preference for anonymous social media (−0.031, 95% C.I. = −0.121 to 0.058, *p* = 0.494). These exceptions illustrated that the effect from vulnerable personality to WSC was fully mediated through negative affectivity and suicide ideation, and preference for using traditional social media other than Weibo may be an independent predictor of WSC. The direct effect from preference for social media other than Weibo was strong (0.363, 95% CI = 0.273 to 0.454, *p* < 0.001). The estimated total, direct, and specific indirect effects from vulnerable personality to WSC of this model are summarized in Table 5. The strongest indirect effect from vulnerable personality to WSC was observed via negative affectivity (0.193, 95% CI = 0.028 to 0.357, *p* = 0.022), followed by negative affectivity and then suicide ideation (0.050, 95% CI = 0.003 to 0.096, *p* = 0.035).

**Table 5:** Total, direct, and indirect effects from vulnerable personality to Weibo suicide communication of Model 3b.

Effects	Estimate	Bootstrap 95% CI	*p*
**Direct effect**	0.085	−0.154, 0.324	0.487
**Specific Indirect effect**			
via suicide ideation	0.027	−0.001, 0.055	0.062
via negative affectivity	0.193	0.028, 0.357	0.022
via anonymous social media preference	−0.011	−0.047, −0.024	0.532
via negative affectivity and suicide ideation	0.050	0.003, 0.096	0.035
**Total indirect effect**	0.258	0.092, 0.423	0.002
**Total effect**	0.343	0.231, 0.455	<0.001

## 4. Discussion

To the best of our knowledge, this is the first study to empirically examine the suicide communication phenomenon on Chinese microblogs and its psychological antecedents. We found that 12.0% of our respondents have exhibited WSC in the 12 months prior to our survey, which is comparable to the prevalence of suicidal ideation in Chinese college students, 10.72% (95% CI: 8.41% to 13.28%) [72]. The result shows that WSC is not a rare phenomenon. More importantly, greater suicide ideation, negative affectivity, neuroticism, and lower agreeableness were found to be correlated with WSC (all *p* < 0.05), which suggests that at-risk users are more likely to talk about suicide. Such a tendency is further supported by the SEM results. Previous studies on offline suicide communication in China found that Chinese suicide victims or attempters often had poor communication skills and often used suicidal behaviors rather than verbal communication when responding to interpersonal conflicts or to express their resistance toward social or family injustice [73,74,75]. In contrast, the use of social media might help some previously hidden suicidal individuals to voice their issues and allow more opportunities for intervention.

On the other hand, the good fit of Model 3b shows that WSC can be well attributed to suicide ideation, negative affectivity, and the preference for using traditional social media, and that the preference for using traditional social media is also significantly correlated with negative affectivity. The results suggest that WSC and the preference for traditional social media, which are both observable behaviors, can be useful signals for detecting negative affectivity and suicide ideation. The model warrants us to take WSC seriously. Even if the person does not commit any self-destructive acts immediately after exhibiting WSC, she/he is still very likely to be suffering from negative emotions or suicide ideation. Broadly reviewing the person’s use of other social media, such as online forums and blogs, would also be helpful to further assess the person’s risk level. If the person expresses suicidal thoughts on more than one social media platform, we would be more confident in raising the concern that s/he may be suffering from suicide ideation or negative affectivity. A sensitive response from lay people should include emotional support and stable companionship. Lay people can also cautiously check whether the person has tried to harm himself/herself previously or has further suicide plans; if so, a referral for professional follow-up is needed.

In Model 3b, the significant correlation between negative affectivity and the preference for using blogs and forums can be interpreted in two possible ways. One is that Weibo users with greater negative affectivity might extend their social media usage to more platforms, such as blogs and forums, and then be more likely to exhibit WSC. In this case, using different social media platforms might increase people’s communication capacities and facilitate them to express themselves more. The other interpretation is that Weibo users who also prefer using other types of social media are more likely to develop negative affectivity and suicidal ideation and further exhibit WSC. The broad use of social media might be associated with symptoms of Internet addiction, which may contribute to depression, anxiety, and even suicidal thoughts and attempts [76,77]. A previous survey in the US also found that the use of blogs and chatrooms was associated with increases in suicidal ideation [78]. In this case, the use of multiple types of social media seems to be a risk factor for negative emotions and suicidal ideation, as well as WSC. In other words, the preference for using traditional social media might be a confounder of negative affectivity and WSC. It seems that the broad use of multiple types of social media brings both opportunities and challenges for suicide prevention. More studies, including longitudinal studies and qualitative studies on individuals with lived experiences of WSC, are needed to investigate which interpretation is applicable, or whether both mechanisms mutually reinforce each other and work in a cycle.

The WSC group and non-WSC group did not report significant differences in using instant messaging, posting to Weibo to friends, and posting to Weibo to the public in our descriptive analysis. The EFA examination of the preference for using social media also found that these three types of social media are clustered together but reported no association with WSC. The results are very likely related to our sample recruitment method: our respondents were all recruited from Weibo, so they might have low variation in using Weibo. In addition, according to the China Internet Network Information Center (CNNIC)’s report, instant messaging is the most popular social media in China with about 89.3% of Internet users in China actively using it. Thus, there might also be low variation in our respondents in using instant messaging. By contrast, online forums and blogs are relatively older social media and their popularity is not as high as instant messaging and Weibo among our respondents. As shown in Table 2, the WSC group reported an average score of using blogs as 1.1 and online forums as 1.0, which indicates occasional use, whereas the non-WSC group reported average scores of 0.6 and 0.4, respectively, which are close to no use. Therefore, our results suggest that the WSC group tends to use more different types of social media than the non-WSC group. The respondents with WSC are also found to be younger and less educated than the non-WSC ones. The lower education level might be related to younger age. In addition, lower education is found to be a risk factor for suicide in China [79]. Future online suicide interventions can pay more attention to people with those characteristics.

In summary, although suicide is still a sensitive topic in China, Weibo users who suffer from negative affectivity or/and suicidal ideation appear to have a higher probability of disclosing their feelings by talking about suicide on Weibo (WSC), especially for those who also often use online forums and blogs for communication. WSC should be considered a sign of suicidal ideation or negative affectivity, which may not be followed by immediate suicidal acts but is certainly a risk factor for suicide. If other social media users, including lay people and online intervention programs, can properly respond when receiving such self-disclosure messages, social media can be transformed into a safety net for suicide prevention. Public education should be organized to encourage online users at risk of suicide to seek help from professionals, and to educate the general users to act as gatekeepers to provide reflective listening, peer support, and referrals for professional follow-up [18].

Some limitations of our study should be noted. The survey sample was not representative of general Weibo users due to the recruitment method. When compared to large sample surveys on social media users, our sample was found to contain more educated males [7,80]. Therefore, we ask for more replicative studies on larger and more representative samples before generalizing our findings to overall Weibo users. Nevertheless, we found there are no gender differences in exhibiting WSC, and age and education level have been controlled for in our model testing. Whether our findings can be applied to users of other social media platforms is subject to further studies. However, if we have to start with one type of social media in China, Weibo is a reasonable choice. The platform has over 500 million users and is popular enough to have users from various backgrounds. Additionally, Weibo users can experience person-to-person communication like instant messaging, person-to-group communication like Facebook, and person-to-public communication like Twitter. The wide spectrum of its functions makes it a representative type of social media. The other limitation is that our study is cross-sectional. We are not certain whether the preference for traditional social media is a confounder of negative affectivity and WSC, or a mediator between the two. Future studies can adapt longitudinal methods to re-examine our proposed models and generate more knowledge on this topic. Qualitative studies on individuals with lived experiences of WSC will also be helpful for scholars to gain more insight into the phenomenon.

## 5. Conclusions

Weibo suicide communication is not a rare phenomenon in China. A similar phenomenon may also occur in other countries, especially those with high penetration rates of social media. Among Weibo users, those with greater negative affectivity or/and suicidal ideation who prefer using blogs and online forums would exhibit WSC more. The capability to disclose their negative thoughts on Weibo opens a door of opportunity for interventions in public health approaches. If they are suffering from negative affectivity, emotional support should be provided to them; if they are having acute suicidal ideation, crisis intervention should be directed to them in a timely manner. Our study adds new knowledge to understanding the situations in which people would talk about suicide in the new media environment. It also provides empirical evidence to justify the usefulness of detecting people at risk by examining online posts, and the significance of developing suicide prevention strategies based on social media, including microblogs, online forums, and blogs.

The best way to predict a potential future public health problem is to prevent it from getting out of hand. The study supports us in considering Weibo users’ talking about suicide as a sign of suicidal ideation or negative affectivity. Mental health and suicide prevention professionals should consider using online social media for detecting and reaching out to people at risk. The general public should be reminded that individuals exhibiting WSC are very likely to be at a certain level of suicide risk. Mocking or expressing indifferent attitudes toward those exhibiting these signs should be avoided. Concluding that such communication is a hoax if the person does not self-harm might be premature. The person might still be suffering from negative affectivity, which is a risk factor for later development of suicidal ideation and behaviors. Early detection of people at risk is critical for early intervention. After learning that someone has talked about suicide on Weibo, broadly examining their posts on various social media platforms might be a promising approach to better assess his/her suicide risk level. Certainly, we also have to be cautious with privacy issues and users’ experiences, and avoid giving the impression that a big brother is watching [1]. Appropriate and sensitive responses and follow-up are critical in preventing people at early risk of suicide from worsening by reducing their negative affect and suicide ideation.
